# Patient's experiences of coughing after lung cancer surgery: A multicenter qualitative study

**DOI:** 10.1002/cam4.6993

**Published:** 2024-02-04

**Authors:** Xiangxi Zhou, Cheng Lei, Xing Wei, Wei Dai, Wei Xu, Yongping Ao, Xianglin Li, Guibin Qiao, Qiuling Shi

**Affiliations:** ^1^ School of Public Health Chongqing Medical University Chongqing China; ^2^ Department of Thoracic Surgery, Sichuan Clinical Research Center for Cancer Sichuan Cancer Hospital & Institute, Sichuan Cancer Center, Affiliated Cancer Hospital of University of Electronic Science and Technology of China Chengdu Sichuan China; ^3^ Department of Thoracic Surgery Guangdong Provincial People's Hospital Guangdong China

**Keywords:** cough, experience, lung cancer, qualitative surgery, study

## Abstract

**Purpose:**

Cough is one of the most common symptoms after lung cancer surgery, which seriously affects the quality of life. Little research has been conducted on patient's experiences of cough following lung surgery. This study aimed to elucidate the experience of coughing after lung cancer surgery from the patient's perspective regarding symptoms and their impacts on daily life, as well as triggers and dealing strategies.

**Methods:**

Between June 2023 and July 2023, we conducted semi‐structured interviews with patients from outpatient clinics of two hospitals who were pathologically diagnosed with lung cancer and experienced cough after surgery through convenience sampling. The interview recordings were transcribed and analyzed by two researchers. The traditional content analysis and thematic analysis were used to identify the common codes, subthemes, and themes.

**Results:**

A total of 28 participants were interviewed. The mean age of the participants was 55.21 years (range: 36–75 years), and 21 participants were female. Most patients (75%) were interviewed within 6 months of surgery. We identified five themes (accompanying symptoms, incentives, effects, solution, and information sources) and 12 subthemes (local symptoms, systemic symptoms, personal factors, external factors, emotion, relationship with others, reduced quality of life, medical measures, nonmedical measures, no measures, relatives and friends, and the Internet). Patients with lung cancer may experience various cough symptoms after surgery, which a variety of internal and external factors can trigger. The coughing imposes a double burden on the physical and psychological due to the negative emotions it provokes.

**Conclusion:**

We generated a concept framework of cough after lung cancer surgery, providing a basis for further development of measurement tools from the patients' perspective. The lack of knowledge related to coughing highlights the need for adequate and timely health education and professional medical care.

## 1 BACKGROUND

Lung cancer is one of the most common cancers threatening human health.^1^ China recorded 871,000 new lung cancer cases and 767,000 deaths, with the second‐highest incidence and highest mortality of lung cancer worldwide.^2^, ^3^ Surgical intervention remains the cornerstone of lung cancer treatment.^4^ Patients with lung cancer may experience a series of postoperative symptoms, commonly including cough.^5^, ^6^ Active cough in patients with effective sputum excretion after surgery is an effective method for lung function recovery.^7^ Studies have shown that 50% of patients have a cough for 1 year, and 25% have a cough lasting more than 5 years after surgery, which is difficult to cure.^8^, ^9^, ^10^

Cough has specific characteristics, such as delayed onset and unproductive cough,^11^ which seriously reduce the quality of life and affect postoperative recovery.^12^ Severe coughing can interrupt sleep, disrupt speech, and reduce quality of life.^13^ The adverse effects of cough include cardiovascular, genitourinary, musculoskeletal, neurological, ophthalmic, psychosocial, respiratory, and cutaneous complications.^14^

Most existing studies have focused on evaluating and treating cough,^15^ with little understanding of cough from the patient's perspective. Several measures to assess a patient's quality of life have been developed,^16^, ^17^ the most effective of which is the Lester Cough Questionnaire (LCQ),^18^ widely used in clinical care. As with many subjective questionnaires, the limited 19‐item LCQ does not fully cover the impact of cough on patients' lives. The iterative nature of item generation begins with domain identification, an inductive process that involves establishing a theoretical foundation of the construct of interest based on the literature, subject matter expert opinion, or qualitative research with the population of interest.^19^ To the best of our knowledge, few qualitative studies focused on patients with cough after lung cancer surgery.

Common treatments recommended for patients with cough include medication and behavioral therapy. Medications include neuromodulators, which are thought to reduce upper respiratory tract sensitivity.^20^, ^21^, ^22^ Behavioral treatments include education on the suspected cause of cough (cough reflex allergy), cough suppression techniques, laryngeal hygiene, and psychoeducational counseling.^23^, ^24^ However, there is still room for improvement in the effectiveness of cough treatments, possibly due to a need for more understanding of the topic. Therefore, it is necessary to conduct qualitative research on cough.

Qualitative research allows patients to report their personal experiences throughout the diagnosis and treatment process,^25^ including their understanding of treatment recommendations and diagnoses and how this process affects their quality of life. Understanding patients' perspectives may enable health care providers to be more supportive. This study aimed to illustrate the experience of coughing after lung cancer surgery from a patient's perspective, which may contribute to a better understanding of this population, thereby enabling the provision of higher‐quality care and a basis for the development of targeted long‐term management.

## 2 METHODS

### 2.1 Design and setting

This multicenter qualitative study was conducted between June and July 2023 in the thoracic surgery department of a specific cancer hospital in Sichuan Province and a tertiary general hospital in Guangdong Province, China. The reason for choosing these two hospitals is that they are located in developed regions (Guangdong Province) and developing regions (Sichuan Province), respectively, so the representativeness of the study subject selection can be increased. Qualitative research was chosen as it can focus on the individual's perceptions of the event phenomenon or experience, show different opinions, and support the development of the questionnaire scale.^26^ The Standards for Reporting Qualitative Research (SRQR) checklist was used to enhance transparency and maintain an audit trail of the study process (Appendix S2).

### 2.2 Participants

The inclusion criteria were patients with (i) age more than or equal to 18 years; (ii) diagnosed with lung cancer through imaging and pathological examination and had undergone pulmonary tumor resection; (iii) complaint of cough during the postoperative recovery period, and assessed by the patient reported cough severity of point 0–10 if the score of 4 or above represents the burden of cough; and (iv) a willingness to participate in the study and had signed an informed consent form.

### 2.3 Sample size estimate

The sample size was determined by reaching “saturation.” The definition of “saturation” in this study was when new data could no longer bring new topics; hence, it was judged to have reached its saturation state. Based on previous studies, we estimated the required sample size for saturation to be 35.^5^

### 2.4 Data collection and management

We screened and recruited patients during postoperative outpatient visits. Potential participants who met the inclusion criteria were asked if they would participate in the study. After the patients agreed to participate in the study, their socio‐demographic and clinical characteristics were collected. At the patient's convenience, interviews were conducted at an outpatient clinic or via phone. These semi‐structured interviews were conducted according to an interview outline (Appendix S1) customized in advance. Each interview was expected to take at least 10 min. The first author (XZ), who had received comprehensive training in qualitative research methodology, had experience in multiple qualitative research studies and had mastered communication skills with patients, conducted all interviews. A notebook was used to record information during the interviews, such as the patient's tone, repetition of specific descriptions, and the interviewer's feelings and thoughts about the current situation. The interview process was recorded with a recording pen, and the audio files were transcribed verbatim into text files within 24 h of the interview. Basic patient information was kept on the real‐world data management platform (https://cdo.epro‐vision.com:81/html/index.html).

### 2.5 Data analysis

Analyzing interview texts obtained is an iterative process. Once the interview audio of the first participant was obtained, data analysis began, which was then carried out alongside data collection in an ongoing process of comparison and iteration. Recordings of patient interviews were transcribed verbatim by two researchers (XZ and CL). The accuracy of the transcripts was verified by two researchers who checked the audiotape content. The traditional content analysis and thematic analysis were used to identify the common codes, subthemes, and themes. First, we generated a codebook by automatically identifying words and manually coding (judgment, labeling, and categorization). The codebook was then updated by adding new words, deleting duplicate words, and combining similar words using an expert panel. Next, two researchers independently coded the same transcripts using the developed codebook. If there were differences in the coding results, a consensus was reached after a discussion with a third researcher. Finally, a comprehensive list of patient‐related items was generated from the qualitative interviews. We summarize the frequency of each code and make a thematic analysis based on it. NVivo 12 (NVivo Qualitative Data Analysis Software; QSR International Pty Ltd., Version 12, 2018) was used for data analysis.^27^, ^28^

### 2.6 Ethical approval

The study was conducted strictly with the principles of the Declaration of Helsinki. This study was approved by the Ethics Committee of Sichuan Cancer Hospital and the Ethics Committee of Guangdong Provincial People's Hospital, China (approval numbers: SCCHEC‐02‐2018‐043 and KY‐Q‐2021‐170‐03). All participants voluntarily participated in the study and could withdraw or retract their data at any time. All the participants received a research information leaflet and signed an informed consent form.

## 3 RESULTS

A total of 28 participants were interviewed (12 in Guangdong and 16 in Sichuan). After interviewing 26 people, the subsequent two interviews yielded no new themes; hence, the data was said to have reached “saturation.” The average interview duration was 15 min (range: 10–24 min), the average age of the patients was 55.21 years (range: 36–75 years), and 21 (75%) were females. Most patients were employees with medical insurance, had a high school and middle school level of education, and were 2 months postoperative. All patients underwent video‐assisted thoracoscopic surgery (Table 1).

**TABLE 1:** Demographic characteristics of the study participants (*n* = 28).

Characteristic	*n*	%
Age, mean (SD), years	55.21 (12.02)	—
Sex		
Male	7	25.00
Female	21	75.00
Education level		
Undergraduate and above	6	21.43
High school and middle school	16	57.14
Primary school or below	6	21.43
Marital status		
Single	1	3.57
Married	26	92.86
Widowed	1	3.57
Medical insurance type		
Resident medical insurance	9	32.14
Employee medical insurance	10	35.72
Rural medical insurance	7	25.00
Others	2	7.14
Employment status		
Employed full time	12	42.86
No job	3	10.71
Retired	6	21.43
Others	7	25.00
Postdischarge time at the interview		
2 weeks to 1 month	4	14.28
1 month to 2 months	5	17.86
2 months to 3 months	8	28.57
3 months to 6 months	4	14.28
6 months to 12 months	3	10.73
Above 12 months	4	14.28

**FIGURE 1:**
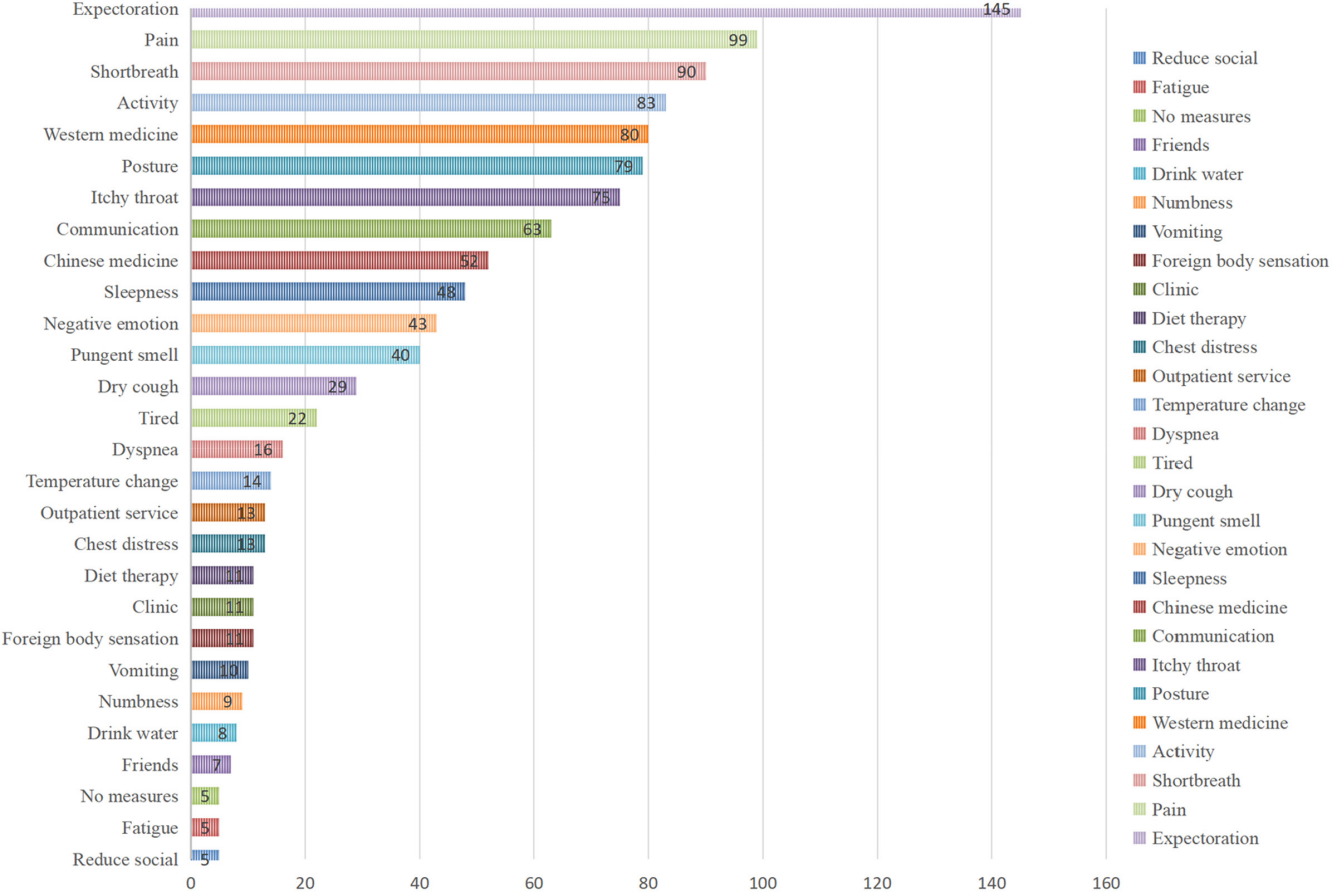
Frequency of cough symptoms in the 28 interviewed patients.

**FIGURE 2:**
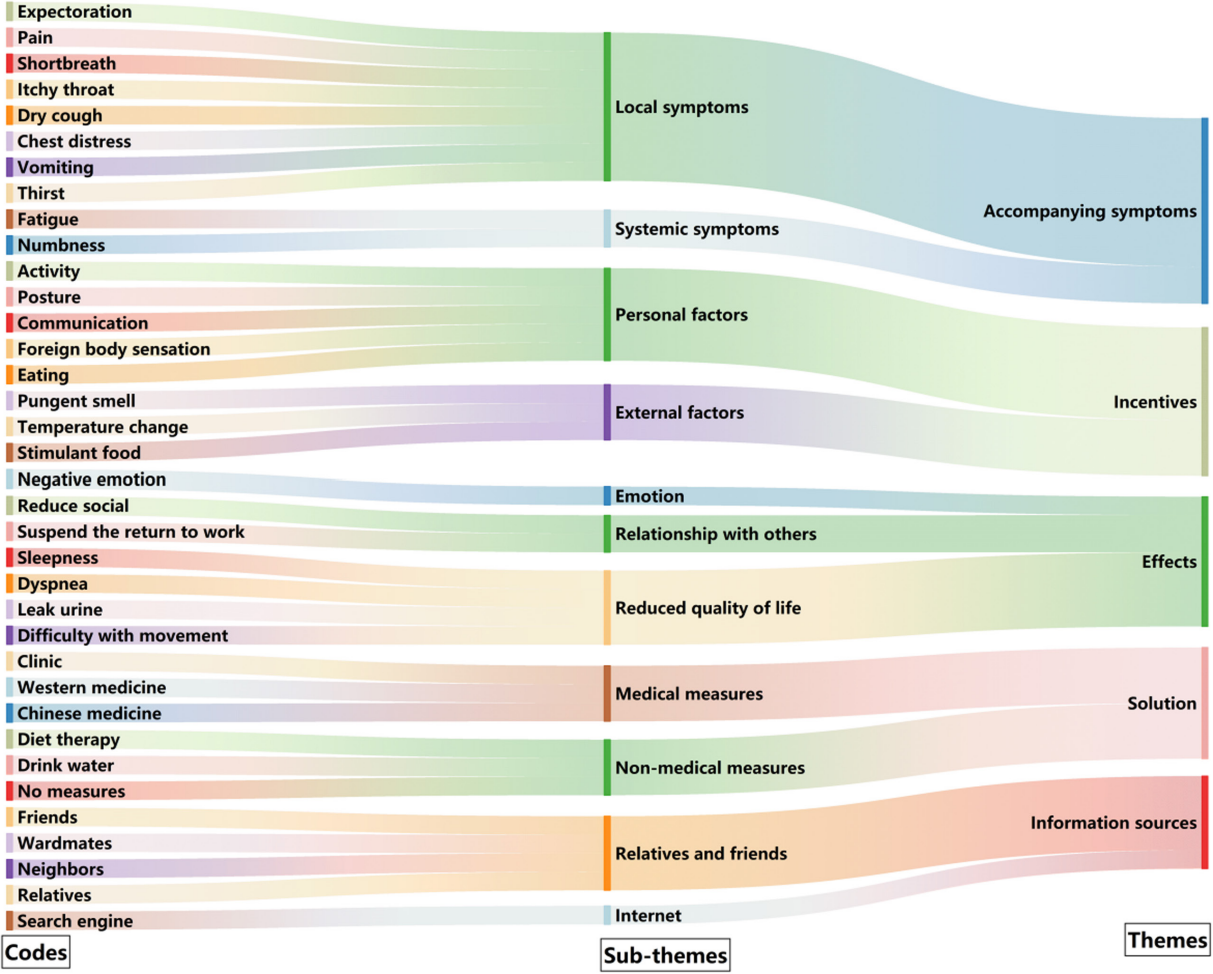
Sample codes and their subordination to each theme.

The 63 codes were aggregated into 12 subthemes, and five themes were identified. A thematic map was used to display the interconnections (Table 2). More than half of the participants mentioned eight codes. The top five codes reported by the patients were: phlegm (145 times, 22 patients), pain (99 times, 16 patients), shortness of breath (90 times, 16 patients), activity (83 times, 17 patients), and Western medicine (80 times, 15 patients) (Figure 1). The process from “codes” to “themes” formation is presented via an alluvial diagram (Figure 2).

**TABLE 2:** Cough experience after lung cancer surgery.

Themes	Subtheme	Example quotes
Accompanying symptoms	Local symptoms	“When I cough, the wound inside will hurt, and then when I cough, I need to use a throw pillow to press down on it to make it less painful.” (Participant 10, female, 69‐year‐old)
		“Sometimes I cough…at first I didn't get sputum, then I got sputum, it's all white and thin sputum.” (Participant 25, male, 74‐year‐old)
		“I cough when I enter an air‐conditioned room, and when the weather changes. Especially when I was cooking and I couldn't breathe because of the fumes, … I felt like I couldn't breathe.” (Participant 23, female, 36‐year‐old)
	Systemic symptoms	“…sometimes I had a pinprick between ribs near the incision. There may be numbness between the breast area and the ribs, but these were intermittent, not persistent.” (Participant 10, female, 69‐year‐old)
		“I felt tired and out of breath when I moved a little. …I feel tired when I sat and talked, …” (Participant 27, male, 64‐year‐old)
Incentives	Personal factors	“Sometimes if I talked too much, I felt cough worse, and then my mouth wound dry out. Anyway it was better to talk less.” (Participant 12, female, 41‐year‐old)
		“I didn't cough when I was lying down. I just coughed a little in the morning after walking around. I've been aching all over since I came home from the hospital.” (Participant 24, female, 56‐year‐old)
		“Especially at night, coughing at night kept me awake, as if there was something stirring in my throat, and then I coughed immediately.” (Participant 22, female, 53‐year‐old)
	External factors	“Especially when I was cooking, and I was cooking and I smelled fumes and I couldn't breathe, and I wanted to get out of the kitchen and I coughed so bad that I fell to the ground.” (Participant 23, female, 36‐year‐old)
		“As soon as I sat down in the air‐conditioned room, I started to cough, and when I coughed, I couldn't be able to bear it…” (Participant 29, female, 56‐year‐old)
		“Whenever the weather changed and my body got a little cold, I wound cough.” (Participant 25, male, 74‐year‐old)
Effects	Emotion	“I was sensitive…When I coughed, I got nervous and had a pinching sensation, which they (daughters) say it was normal.” (Participant 10, female, 69‐year‐old)
		“I didn't have any extra ideas, I'm a cheerful person…I didn't think I could do anything with this disease…” (Participant 22, female, 53‐year‐old)
		“The cough affected me, I was very afraid, I wonder if there was any special medicine can you give me to eat, I was afraid that the cough made me die…” (Participant 23, female, 36‐year‐old)
	Relationship with others	“The cough wass very annoying, and sometimes I felt that it was not convenient to talk…life was very inconvenient.” (Participant20, female, 49‐year‐old)
		“Last month when I went to work, I climbed the stairs and terrified my colleagues … Now my boss asked me to take time off to recover from illness and not to go to work.” (Participant 23, female, 36‐year‐old)
	Reduced quality of life	“Sometimes I was leaking urine because I coughed. I couldn't breathe when I had a continuous cough. I felt like I'm dying.” (Participant20, female, 49‐year‐old)
		“The cough had always been like this, and sometimes I couldn't sleep at night.” (Participant 11, female, 43‐year‐old)
Solution	Medical measures	“After coughing for a while, I took some traditional Chinese medicine and the cough disappeared.” (Participant 7, male, 66‐year‐old)
		“… Later, I asked the doctor to prescribe two bottles of cough syrup. After taking the syrup, the cough disappeared and I didn't care it.” (Participant 30, female, 40‐year‐old)
	Nonmedical measures	“…when I coughed I drunk more water. I felt that drinking water could alleviate the symptoms…” (Participant 19, female, 61‐year‐old)
		“My mother is a doctor, and she once heard that I can eat white radish to cure a cough. I ate it for a while and the cough went away.” (Participant 18, female, 70‐year‐old)
	No measures	“In fact, I did not do much, because I thought cough is a self‐healing process, I just let it come out naturally.” (Participant 13, female, 48‐year‐old)
Information sources	Relatives and friends	“…The last time I met a friend, I told him about my condition. He said that my condition was normal, so I never came back to see a doctor.” (Participant 14, female, 59‐year‐old)
		“I have a friend who had this surgery two years ago…She and another colleague coughed for two or three months, so I have this perception.” (Participant 13, female, 48‐year‐old)
	Internet	“…I didn't know anything about cough after surgery, and the doctor didn't tell me, so I had to check everything online by myself.” (Participant 12, female, 41‐year‐old)

### 3.1 Accompanying symptoms

Local and systemic symptoms often accompany the onset of cough, and patients' perceptions of symptoms vary. Local symptoms are mainly concentrated in the throat and respiratory tract. In contrast, systemic symptoms primarily affect the cardiovascular, cerebrovascular, and nervous systems. Patients with systemic symptoms may have an exaggerated perception of the degree of the description.

#### 3.1.1 Local symptoms

The local symptoms reported by patients include expectoration, pain, shortness of breath, itchy throat, vomiting, and dry mouth. Most patients reported coughing phlegm, predominantly white or yellow sputum. Many patients reported that coughing was accompanied by pain and shortness of breath, while half reported a dry cough. It is worth noting that the pain associated with coughing varies between patients.

#### 3.1.2 Systemic symptoms

Systemic symptoms include fatigue and numbness, mainly observed in patients with severe disease. Some patients specifically mentioned feeling very tired when they coughed. This fatigue is mainly due to the weakness and hypoxia caused by excessive force during coughing because the patient has too many lung lobes removed or his/her lung function has not fully recovered after surgery. Numbness is caused by nerve damage due to the wound. Compared with the resting state, most patients reported a constant feeling of coughing.

### 3.2 Incentives

Coughing is typically caused by stimulating factors. The most common reasons for coughing were inherent; however, external factors caused or aggravated some. When the irritants disappeared, the patient's cough resolved. Some patients reported that they could not cough for long without exposure to personal factors. Furthermore, changes in external factors had different effects on patient's coughs.

#### 3.2.1 Personal factors

Speaking, various postures, movements, walking, and the sensation of foreign bodies can cause cough to appear or worsen. More than half of the patients mentioned that different postures caused changes in their cough. Most said that talking, movement, or walking worsened their cough.

#### 3.2.2 External factors

Temperature changes (dramatic weather changes, entering air‐conditioned rooms) and irritating odors (kitchen fumes and second‐hand smoke) can also cause coughing. A third of the patients experienced irritating odors (kitchen fumes and second‐hand smoke). A few patients thought climate change caused their cough. Irritating food had varying effects on patients: some said irritating food led to cough aggravation. There were also differences in coughing according to the time of day, with some patients reporting similar levels of coughing during the day and night. In contrast, others reported significantly higher levels of coughing at night than during the day.

### 3.3 Effects

The effects of coughing on patients mainly manifest as negative emotions, weakened relationships with others, and reduced quality of life. Negative emotions mainly arise from oneself and others and include annoyance, worry, fear, depression, nervousness, anxiety, sensitivity, and embarrassment. Reducing social contact and delaying return to work can delay a patient's return to social life, thereby weakening their relationships with other people. The reduced quality of life was mainly due to poor sleep quality at night, fatigue, and difficulty moving and breathing. Some female patients also experienced urinary incontinence during coughing.

#### 3.3.1 Emotion

More than half reported negative emotions due to coughing, causing them to become over‐self‐conscious and putting psychological pressure on their families. A few patients said that the cough did not affect them and did not worry about it.

#### 3.3.2 Relationship with others

Patients reduced social activities and suspended work, affecting their relationships and reducing their frequency of communication. A few patients said they had suspended work because of their cough, which reduced their social interactions. These patients had more severe symptoms than others.

#### 3.3.3 Reduced quality of life

Coughing reduces quality of life by causing night‐time sleep disruption, fatigue, urinary incontinence, and breathing difficulty. Participants reported that it interrupted their night‐time sleep, causing them to either wake up repeatedly during the night or be unable to sleep. Cough leading to hypoxia and breathing difficulties is a typical condition, and recovery from this condition takes time. Females reported urinary incontinence when coughing intensely, primarily in older women with loose pelvic floor muscles.

### 3.4 Solution

When coughing occurs, respondents generally use positive coping measures, including medical and nonmedical measures. However, all patients reported that they were ineffective even if they took appropriate measures; hence, they may need more support to relieve their symptoms and recover. There were also a small number of patients who thought the cough was normal and let it develop naturally.

#### 3.4.1 Medical measures

Medical measures mainly consisted of seeking medical attention and taking Chinese and Western medicines. The majority explicitly mentioned that they had sought medical help for coughing. More than half said they were taking Western medicine. Some mentioned that they had tried to control their cough with traditional Chinese medicine.

#### 3.4.2 Nonmedical measures

Nonmedical measures included drinking water and dietary intake. Patients reported drinking water to control their cough and had tried dietary therapy. Dietary therapy is auxiliary in relieving coughs and is often combined with Chinese or Western medicine.

#### 3.4.3 No measures

Few patients did not take any measures to control their cough after the operation and believed that coughing was a natural process that did not require medication or human intervention.

### 3.5 Information sources

To further understand cough symptoms and solutions, patients mainly communicated with relatives, friends, and ward mates, with one patient searching for cough‐related knowledge and solutions online. As cough is one of the most unpleasant symptoms after lung cancer surgery, patients may need more health education from medical staff after discharge to help them smoothly transition through the postoperative recovery period.

#### 3.5.1 Relatives and friends

Patients not only learn about cough‐related knowledge and solutions through their relatives and friends but are also willing to learn through friends of friends. Some said they discussed coughing with their fellow patients in the ward, acquired cough knowledge and solutions from their friends and relatives, and sought solutions through exchanges with neighbors.

#### 3.5.2 Internet

Only one patient explicitly stated that she had searched the Internet for cough‐related information and that the knowledge provided by the medical staff did not meet her needs.

## 4 DISCUSSION

We identified a framework for patients' cough experience after lung cancer surgery, which included five themes: symptoms, triggers, effects, solutions, and sources of information. Cough‐related symptoms can affect the mood and quality of life of patients. Simultaneously, female respondents tend to have more severe symptoms and more negative emotions than male patients. Postoperative cough after lung cancer surgery lacks standardized management and systematic health education. Hence, this study may fill the gap in the literature regarding patient perceptions of postoperative cough.

We found that patients with coughing have different degrees of negative emotions, which impact their quality of life, depending on their sex. Females surveyed tended to have higher levels of anxiety and worse quality of life than males, which is similar to other studies.^29^ A study in Barcelona found that females had higher rates of anxiety, depression, and poorer health than males.^30^ This may be because females are more sensitive and pay more attention to body changes than males.^31^, ^32^ This suggests that medical staff should consider the sex differences between male and female patients when providing personalized nursing and psychological support for female patients to improve their overall health.

Laryngeal discomfort was reported by most participants in this study, with symptoms including expectoration, itchy throat, and breathlessness; moreover, they reported pain, fatigue, and other symptoms. The occurrence of these concomitant symptoms indicates that patients experience a heavy burden of symptoms after surgery. Some studies have shown that more than half of patients have multiple moderate or severe symptoms after surgery, and major cancer surgery leads to a higher burden of readmission with more comorbidities.^33^, ^34^ It is necessary for health care providers to consider a standardized management of symptoms after surgery.^35^ Electronic health record systems and patient‐reported outcomes are widely used in some countries to standardize patient management after discharge.^36^, ^37^ Therefore, solutions based on digital health care management are expected to be better.^38^ Future management of cough patients should consider higher levels of supportive interventions to meet patient needs and improve patient satisfaction.

We found that few patients had received adequate knowledge about coughing from medical staff. Moreover, the majority needed more awareness. Some patients mentioned that health care providers were not meeting their needs for care and support, leading to confusion during treatment and postoperative recovery. It may be due to the large number of patients and operation volumes in tertiary hospitals, resulting in a relative shortage of medical personnel and low satisfaction.^39^ Patients with low literacy, poor cooperation, and poor compliance cannot receive adequate, timely health education. The medical staff needs to conduct preoperative and postoperative health education for patients.^40^, ^41^ Patients urgently require continuous attention from medical professionals after discharge. It may be beneficial to provide cough‐related information to recovering patients at home.^42^, ^43^

We found that most patients took measures to treat their cough; however, these treatments were generally ineffective for some patients. Although some participants reported benefits from prescription drugs and traditional Chinese medicine, no medication eliminated their cough and cough‐related symptoms. Hence, there may be no specific medicines suitable for treating postoperative cough. Studies have shown that cough is related to postoperative bronchial morphological changes, and the effect of medication control is often unsatisfactory.^44^, ^45^ This suggests that the current methods for treating postoperative cough have many limitations, and there is a need to explore the causes of cough after lung cancer surgery to enable the provision of patient‐targeted interventions.

### 4.1 Limitation

Our study had several limitations. Firstly, although we recruited participants from hospitals of two different economic development levels to enrich the representation, bias by convenience sampling could not be avoided, and the results should be treated cautiously. Secondly, we focused on the cough experiences of patients with lung cancer. None of the respondents considered whether they got a cold, flu, or COVID‐19 after surgery. These factors may also cause cough symptoms in patients, which may cause a certain lack of information for constructing the experience framework. Finally, most respondents describe their experiences as recollective, which may lead to recall bias in the data. Future studies should consider additional factors and build a more comprehensive framework to elucidate complex cough‐related relationships.

## 5 CONCLUSIONS

Our study provides insights into the qualitative perspectives of patients with cough after lung cancer surgery. Our findings generate a conceptual framework for postsurgery cough that provides a basis for further developing patient‐reported measurement tools. We appeal to health care providers to offer personalized health education to lung cancer patients after surgery to improve their cough knowledge and quality of life.

## AUTHOR CONTRIBUTIONS

**Xiangxi Zhou:** Conceptualization (equal); data curation (equal); formal analysis (equal); funding acquisition (equal); investigation (equal); methodology (equal); resources (equal); software (equal); supervision (equal); writing – original draft (equal); writing – review and editing (equal). **Cheng Lei:** Conceptualization (equal); data curation (equal); formal analysis (equal); funding acquisition (equal); investigation (equal); methodology (equal); resources (equal); supervision (equal); validation (equal); writing – original draft (equal); writing – review and editing (equal). **Xing Wei:** Conceptualization (equal); data curation (equal); methodology (equal); project administration (equal); supervision (equal); writing – original draft (equal); writing – review and editing (equal). **Wei Dai:** Conceptualization (equal); data curation (equal); methodology (equal); project administration (equal); supervision (equal); writing – original draft (equal); writing – review and editing (equal). **Wei Xu:** Data curation (equal); formal analysis (equal); methodology (equal); writing – review and editing (equal). **Yongping Ao:** Data curation (equal); formal analysis (equal); methodology (equal); writing – review and editing (equal). **Xianglin Li:** Data curation (equal); formal analysis (equal); methodology (equal); writing – review and editing (equal). **Guibin Qiao:** Conceptualization (equal); funding acquisition (equal); investigation (equal); methodology (equal); supervision (equal); writing – review and editing (equal). **Qiuling Shi:** Conceptualization (equal); funding acquisition (equal); methodology (equal); project administration (equal); supervision (equal); writing – review and editing (equal).

## FUNDING INFORMATION

This work was supported by the National Key R&D Plan for Intergovernmental Cooperation, the Ministry of Science and Technology of China [2022YFE0133100].

## CONFLICT OF INTEREST STATEMENT

None.

## Supporting information


Appendix S1

Appendix S2

## Data Availability

The dataset used and analyzed during the current study is not publicly available due to the sensitivity of the data (transcribed interviews from patients). It may be available from the corresponding author upon reasonable request.
