# Supplementary material for: Patient's experiences of coughing after lung cancer surgery: A multicenter qualitative study
Source: Cancer Med. 2024 Feb 4;13(2):e6993. doi: 10.1002/cam4.6993 (PMC10839156; doi:10.1002/cam4.6993)
Supplement: Supplementary file 1 — Appendix S1 [file CAM4-13-e6993-s002.docx]

Appendix S1. Questions for the semi-structured interview

When did you start coughing after surgery?

How long have you been coughing now?

How do you feel when you cough?

Do you produce phlegm when you cough? What kind of phlegm is it?

Did you have a fever when you were coughing?

Has your cough changed over time? What changes? What's different now than before?

How often does the cough occur? When is it more frequent?

Have you recently been infected with COVID-19? How long have you been infected?

Can you control your cough?

Does coughing in certain body positions make it worse? (eg: Sit up, lie flat)

Are there other factors that can make your cough better or worse? What is it? (eg: Smoking, second-hand smoke)

How does coughing affect your daily life?

How do you feel when you cough in front of people?

Is the cough painful to you? How painful is it?

What thoughts or concerns do you have when you cough?

How do you deal with coughing?

Where did you get help? How did you do it?
